# 

**Published:** 1905-04

**Authors:** 


### CONTENTS.

ORIGINAL COMMUNICATIONS—

Broncho-Pneumonia in Children. By Louis C. Rouglin, M.D. .......... 1
An Interesting Case of Lightning Stroke. By N. C. Mitra, M.A., M.B., Ranchi, Bengal, India... 7
Therapeutic Application of Anesthesin in Medicine. By Dr. Honigschmied, Practicing Physician at Weistrach .......... 10

CORRESPONDENCE—

Some Facts Regarding Reorganization in Other States .......... 15

SOICETY REPORTS—

Clinical Society of the New York Polyclinic Medical School and Hospital .......... 21

EDITORIAL NOTES AND COMMENTS—

"Hand-Fixing." .......... 32
De Senectute .......... 35
Trouble at the Grady Hospital .......... 36
Meeting of the Medical Association Gof eorgia .......... 37
Proposed New Constitution and By-Laws. (Concluded from March issue.) .......... 39

NEWS AND NOTES .......... 47

CONTENTS CONTINUED .......... X

